# Multimodality Imaging to Predict Response to Systemic Treatment in Patients with Advanced Colorectal Cancer

**DOI:** 10.1371/journal.pone.0120823

**Published:** 2015-04-01

**Authors:** Linda Heijmen, Edwin E. G. W. ter Voert, Wim J. G. Oyen, Cornelis J. A. Punt, Dick Johan van Spronsen, Arend Heerschap, Lioe-Fee de Geus-Oei, Hanneke W. M. van Laarhoven

**Affiliations:** 1 Department of Medical Oncology, Radboud University Medical Center, Nijmegen, the Netherlands; 2 Department of Radiology and Nuclear Medicine, Radboud University Medical Center, Nijmegen, the Netherlands; 3 Department of Medical Oncology, Academic Medical Center, University of Amsterdam, Amsterdam, the Netherlands; 4 Department of Medical Oncology, Canisius Wilhelmina Hospital, Nijmegen, the Netherlands; 5 MIRA Institute for Biomedical Technology and Technical Medicine, University of Twente, Enschede, The Netherlands; University General Hospital of Heraklion and Laboratory of Tumor Cell Biology, School of Medicine, University of Crete, GREECE

## Abstract

**Aim:**

Aim of this study was to investigate the potential of ^18^F-FDG PET, diffusion weighted imaging (DWI) and susceptibility-weighted (T2*) MRI to predict response to systemic treatment in patients with colorectal liver metastases. The predictive values of pretreatment measurements and of early changes one week after start of therapy, were evaluated.

**Methods:**

Imaging was performed prior to and one week after start of first line chemotherapy in 39 patients with colorectal liver metastases. ^18^F-FDG PET scans were performed on a PET/CT scanner and DWI and T2* were performed on a 1.5T MR scanner. The maximum standardized uptake values (SUV), total lesion glycolysis (TLG), apparent diffusion coefficient (ADC) and T2* value were assessed in the same lesions. Up to 5 liver metastases per patient were analyzed. Outcome measures were progression free survival (PFS), overall survival (OS) and size response.

**Results:**

Pretreatment, high SUV_max_, high TLG, low ADC and high T2* were associated with a shorter OS. Low pretreatment ADC value was associated with shorter PFS. After 1 week a significant drop in SUV_max_ and rise in ADC were observed. The drop in SUV was correlated with the rise in ADC (r=-0.58, p=0.002). Neither change in ADC nor in SUV was predictive of PFS or OS. T2* did not significantly change after start of treatment.

**Conclusion:**

Pretreatment SUV_max_, TLG, ADC, and T2* values in colorectal liver metastases are predictive of patient outcome. Despite sensitivity of DWI and ^18^F-FDG PET for early treatment effects, change in these parameters was not predictive of long term outcome.

## Introduction

The liver is the most frequently affected organ in disseminated colorectal cancer and most patients with metastatic disease rely on palliative systemic treatment [1]. Depending on the schedule, response is commonly evaluated 8–9 weeks after start of treatment. Currently, response to treatment is monitored by size response evaluation according to the RECIST criteria [2]. A reliable tool that predicts response early after start of therapy is desirable, as this would prevent unnecessary toxicity and costs.

Size response evaluation may be a suboptimal method to assess efficacy of targeted therapies, since necrosis or fibrosis without reduction in tumor size may occur [3,4]. The outcome of palliative systemic therapy has improved in recent years by the addition of targeted agents to cytotoxic treatment. The addition of bevacizumab, a monoclonal antibody which targets the vascular endothelial growth factor has resulted in significant benefits in progression free survival and overall survival [5].

Several functional imaging techniques have shown potential for prediction of response as well as early response monitoring. In a systematic review, the role of ^18^F-FDG PET in treatment response prediction and monitoring in advanced stage colorectal cancer was evaluated. Four out of five studies showed changes in standardized uptake values (SUV) of ^18^F-FDG PET after start of systemic treatment [6] and two of these four studies showed a correlation with long term outcome [7,8]. In the palliative setting, metabolic response after one cycle of chemotherapy was a stronger predictor for survival than RECIST evaluation after 3 cycles of chemotherapy [9]. However, combined with other functional imaging techniques ^18^F-FDG-PET might be an earlier and stronger predictor of response to therapy.

Diffusion weighted imaging (DWI) is another promising functional imaging tool for tissue characterization, pretreatment response prediction and response evaluation in cancer [10,11]. From the diffusion weighted MR images an apparent diffusion coefficient (ADC) can be calculated, which is a measure for the mobility of water. The ADC is inversely correlated to the cell density, since cellular membranes inhibit water movement [12]. Changes in ADC may precede changes in tumor size, since early after start of treatment changes in cellularity and necrosis may already occur. It has been demonstrated that ADC increased within days after chemotherapy in colorectal cancer metastases [13,14]. These studies investigated the correlation between ADC change and response in metastases. However, to be implemented in clinic, changes in ADC should predict response or (progression free) survival in the patient.

Vascular changes induced by bevacizumab and chemotherapeutic treatment may also be indicative of response. Furthermore, vascular status by itself may predict response, since hypoxia and vascular supply of the chemotherapy influence treatment efficacy. T2* MRI, also called intrinsic susceptibility-weighted MRI or BOLD, is investigated as an alternative to (dynamic) contrast enhanced imaging [15,16]. The transverse relaxation time, T2*, is dependent on magnetic field inhomogeneity and is therefore significantly affected by the presence of paramagnetic deoxyhemoglobin. The higher the deoxyhemoglobin concentration, the lower the T2* value [17]. To our knowledge, thus far no studies using T2* for response prediction of metastatic colorectal cancer have been published.

The aim of this prospective study was to assess the predictive value of ^18^F-FDG PET, DWI and T2* MRI for response to first line chemotherapy and outcome in advanced stage colorectal cancer patients.

## Material and methods

### Patients

Between August 2009 and September 2012 patients of 18 years and older with histologically confirmed colorectal cancer and non-resectable colorectal liver metastases starting first line palliative or neoadjuvant chemotherapy were approached for participation in this study. Exclusion criteria were: Karnofsky performance status <70, (adjuvant) chemotherapy <6 months before study participation, renal function impairment (MDRD <60 ml/min/1.73 m2) or pregnancy. Specific contra-indications for ^18^F-FDG PET (including diabetes mellitus) or MRI only excluded patients for either the PET or MRI part of the study protocol. The study was approved by the medical ethics committee (Commissie Mensgebonden Onderzoek regio Arnhem-Nijmegen: approval number: 2008/194). All patients provided written informed consent before entering the study.

### Imaging methods

Before start of chemotherapy ^18^F-FDG PET, contrast CT and MRI were performed. These scans were performed median 5 days before start of treatment. One week after start of chemotherapy (range 6–8 days), ^18^F-FDG PET and MRI were repeated. After 3 cycles of chemotherapy an ^18^F-FDG PET and contrast CT were performed to assess treatment response.

PET CT-scans were performed on a PET/CT scanner: a Biograph Duo (Siemens Medical Solutions USA, Inc., Knoxville, TN, USA) for the first 26 patients and a Biograph mCT PET/CT scanner for the 13 most recently enrolled patients. Both PET/CT-scanners were accredited by the EANM-EARL QA/QC program for quantitative PET [18]. Images of the Biograph Duo scanner were reconstructed using the 4 iterations/16 subsets (4i/16s) OSEM-2D reconstruction algorithm, smoothed with a 5-mm FWHM Gaussian filter. Images of the Biograph mCT scanner were reconstructed using TrueX, time of flight and the 3i/21s reconstruction algorithm, smoothed with an 8-mm FWHM Gaussian filter. All PET scans were corrected with a low dose CT for attenuation correction [19].

Contrast enhanced CT using ioversol (125ml Optiray 350) was performed. Scanning parameters for contrast-enhanced CT imaging were care dose referenced at 80 mAs for the thorax and 130mAs for the abdomen, 110 kV, rotation time 0.8s, slice 3 mm, slice collimation 2.5mm, feed/rotation 10mm for thorax and 6.3mm for the abdomen on the Biograph duo. A delay of 40s after injection of ioversol was applied for thorax imaging and a delay of 10s was set to automatically shift to the abdominal imaging. The scanning parameters for CT on the Biograph mCT were set as follows: 200 mAs, 120 kV, pitch 1.2, slice 1 mm, delay of 40s after contrast injection and bolus tracking.

DWI and gradient echo measurements to obtain ADC and T2* values were performed on a 1.5T MR system (Magnetom Avanto, Siemens Medical Solutions, Erlangen, Germany) using a body coil for excitation and a body matrix coil combined with spine matrix coil for signal reception. DWI was performed with an EPI sequence and diffusion weighted images were obtained in three orthogonal directions with b-values of 50, 300, and 600 s/mm2. A 2D- Prospective Acquisition CorrEction navigator triggering was used to avoid respiratory motion artifacts. Parallel imaging was combined with Generalized Autocalibrating Partially Parallel Acquisition and an acceleration factor of 2. Spectrally Adiabatic Inversion Recovery was included to suppress the fat signal. Other scan parameters were as follows: TR 2000 ms; TE 82 ms; 30 transversal slices of 6.0 mm thickness separated by 1.2 mm; field of view 400x400 mm; matrix size 192x192; bandwidth 1736 Hz/px; 3 averages; anterior-posterior phase encoding direction.

To obtain T2* images a spoiled gradient-recalled echo, FLASH 2D, sequence was employed. Each slice was obtained with multiple TE values (4.76, 9.53, 14.29, 19.06, 23.82, 28.58, 33.35, 38.11, 42.88, 47.64, 52.40 ms) and a TR of 225 ms. Other parameters were as follows: flip angle 25 degrees; field of view 400x400mm; slice thickness 6.0 mm; matrix size 128x128. Parallel acquisition (GRAPPA) with an acceleration factor of 2 was used. Patients continued normal breathing during the T2* scans.

### Image analysis

The maximum and mean SUV (SUV_max_ and SUV_mean_) of the liver lesions with a maximum of 5 liver lesions per patient were assessed by applying a variable threshold delineation method based on the signal-to-background ratio [20]. The liver lesions were selected based on good visible separation from other metastases and the right kidney on the PET images. The SUV was corrected for total body weight. The adaptive threshold was calculated with the formula: *threshold* = *SUVbackground*+0.41*(*SUVmax-SUVbackground*)[21].

All tumors were delineated using Inveon Research Workplace (IRW 3.0, Siemens Medical Solutions, USA). A volume weighted average of the SUV_max_ over the assessed liver lesions was calculated to have one average SUV_max_ for each patient. A total lesion glycolysis (TLG) was calculated by multiplying the metabolic volume with the SUV_mean_ for each metastasis. The sum of the total lesion glycolysis (TLG) of the assessed tumors was calculated, to have one TLG value per patient.

The ADC images were calculated automatically by the Syngo VB17 scanner software using a noise level of 10. ROI’s were manually drawn on the diffusion weighted b = 50s/mm2 images for optimal tumor-background contrast and were drawn around the entire metastasis (on all slices). ROI’s were drawn around the same lesions as delineated on the PET scans. Inveon Research Workplace overlays the ADC map on diffusion weighted b = 50s/mm2 image. The mean ADC value in each metastasis was calculated.

T2* maps were generated using in-house built software based on Matlab (MathWorks, Natick, MA, USA). The echo time data was fitted pixel by pixel to a mono-exponential curve. ROIs drawn on diffusion weighted b = 50s/mm2 image were copied on the T2* map, to calculate the mean T2* values. Baseline images and the images one week after start of treatment were analyzed by before the outcome after 3 cycles was evaluated. To evaluate CT response, the maximum lesion diameters after 3 and 6 cycles were compared to lesion size before start of treatment.

### Statistics

The following outcomes are indicative of the prognostic performance that can be demonstrated with a power of approximately 80% and an overall alpha of 0.05 for different assumptions regarding the hazard ratio (HR) and standard deviation (SD) of the imaging parameter. In case of high prognostic performance (HR = 0.8) of the imaging parameter, the standard deviation needs to be at least 2, for 30 patients per group to be sufficient. For a HR of 0.90, 30 patients per group are sufficient if SD is at least 4.25, whereas 40 patients are required to demonstrate this association with an SD of 3.75. If the prognostic performance is even poorer (HR 0.95), 40 patients are sufficient if the SD is at least 7.5 and 60 if SD is 6. In total, 39 patients were enrolled in our study. Therefore, this study can demonstrate the prognostic value of an imaging parameter, if the imaging parameter has a high prognostic performance.

All data acquired were imported in SPSS 20.0 (IBM SPSS Statistic, SPSS Inc., IBM, Chicago, IL). To assess the predictive value of the pretreatment SUV_max_, TLG, ADC and T2* measurement to progression free and overall survival a Cox proportional regression analysis was used. A multivariate Cox proportional regression analysis was used to assess predictive value of combined parameters. Given the limited number of patients in this study, no multivariate analysis to correct for (other) prognostic parameters was performed. Progression free survival was defined as the time from start of therapy to disease progression on CT (defined according to RECIST criteria) or death by any cause. Overall survival was defined as time from start of treatment to death by any cause.

A paired sample T-test was performed to assess whether the measured SUV, T2* and ADC one week after start of therapy significantly differed from the values before start of therapy. Cox regression analysis was performed to assess the predictive value for PFS and OS, if the parameters showed a statistically significant change after a week.

Furthermore, we assessed the correlation between the pretreatment values and early changes on PET and MRI with the changes on CT after 3 cycles of chemotherapy in a lesion-by-lesion and patient-by-patient analysis.

## Results

### Patients

Thirty-nine patients participated in the study, 28 males and 11 females (mean age 62 years, range 29–77 years). In total, 154 liver metastases were assessed. Patient characteristics are summarized in Table 1. Two patients underwent MRI only. In two patients only an ^18^F-FDG PET/CT was performed. The first follow-up scan (one week after start of treatment) was not completed by eight patients. Side effects of chemotherapy was the main reason for protocol deviations. The final follow-up scans (after 3 treatment cycles) were not completed by 7 patients,Table 2. Median follow-up was 65.5 weeks. After three cycles 43.6% (N = 17) of the patients showed partial response, 41.0% (N = 18) of the patients had stable disease and 12.8% (N = 5) of the patients died or had severe progression clinically.  

**Table 1:** Characteristics of participating patients.

Patient characteristics	*N* = 39
**Sex**	71.8% male (*N* = 28)
	28.2% female (*N* = 11)
**Mean age**	62 years (range 29–77)
**Localization primary tumor**	41.0% Right hemicolon (*N* = 16)
	28.2% Left hemicolon, including sigmoid (*N* = 11)
	23.1% Rectal cancer (*N* = 9)
	7.7% Unknown location in colon (*N* = 3)
**Treatment**	CAPOX+ bevacizumab (*N* = 32)
	Capecitabine+ bevacizumab (*N* = 4)
	CAPOX (*N* = 1)
	UFT (*N* = 1)
	FOLFOX with hyperthermia (*N* = 1)
**Intention of treatment**	Neoadjuvant (*N* = 5)
	Palliative (*N* = 35

CAPOX = capecitabine and oxaliplatin; UFT = tegafur-uracil; FOLFOX = fluorouracil and oxaliplatin

**Table 2:** Average ^18^F-FDG PET, MRI and CT parameters at pretreatment, one week after start of therapy and after 3 cycles of therapy.

	Pretreatment Average (SD)	1 week Average (SD)	3 cycles Average (SD)
**FDG PET**	*N* = 37	*N* = 30	*N* = 29
** SUVmax (g/cm3)**	11.4 (4.7)	8.8 (3.7)	5.0 (3.3)
** TLG (g)**	1084 (1244)	726 (1145)	144 (212)
**MRI**	*N* = 36	*N* = 29	
** DWI (ADC)**	1.21 (0.17)	1.27 (0.20)	
T2* (ms)	30.2 (7.0)	26.7 (8.1)	
**CT (RECIST)**	*N* = 39		*N* = 31
** Sum of diameters (mm)**	153 (90)		104 (74)

### Predictive value of pretreatment ^18^F-FDG PET and MRI parameters

**Fig 1:**
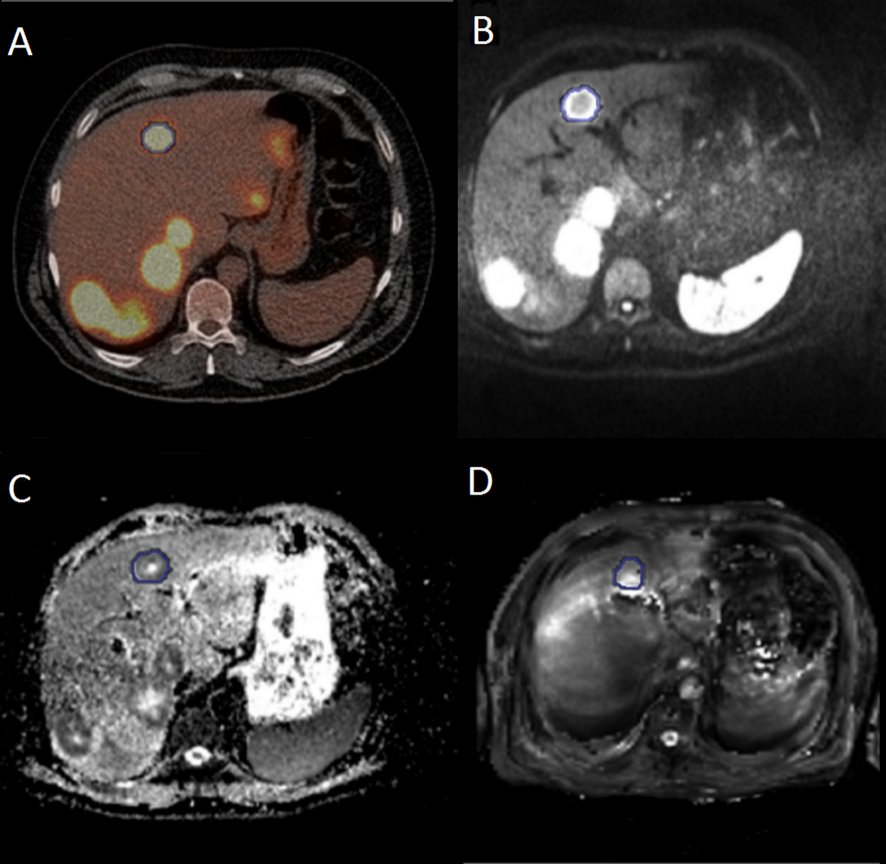
Typical images acquired with FDG PET, DWI and T2* of a patient with multiple liver metastases . One liver metastasis is delineated with a blue line on A) FDG-PET/CT B) a diffusion weighted image (b-value 50 s/mm2), C) ADC-map and D) T2*-map.

An example of a pretreatment ^18^F-FDG PET, DWI, ADC map and T2* image of a patient with multiple liver metastases is shown in Fig. 1. In general, high uptake of FDG was visible in the liver metastases on the PET-images. On the ADC-map, there was a dark signal (low ADC) in the rim of all tumors and high signal in the centre of most tumors, indicating central necrosis. There was little contrast between liver and tumor on the T2*-map.

The average SUV_max_ in the liver lesions was 11.3 g/cm3 (range 5.8–20.5 g/cm3) and the mean sum of the TLG in the assessed liver lesions was 1084g (range 12.8-4845g). The SUV_max_ value was not a significant predictor of PFS, but was predictive of OS. Each 1.0 increase in SUV_max_ was associated with a 1.125 higher risk of death (95% CI 1.020–1.241, p = 0.02). Although TLG was not predictive of PFS, it did show predictive value for OS: each 100g increase in pretreatment TLG value was associated with a 1.047 increased hazard of dying (95% BI 1.010–1.085, p = 0.01).

**Fig 2:**
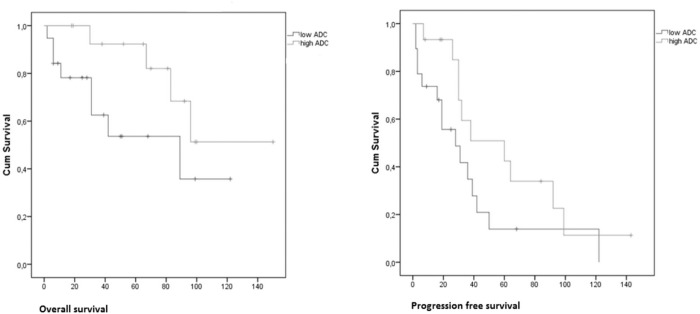
Kaplan Meier Survival curve for a higher (green) or lower (blue) than mean ADC value (1.21 mm2/s) at pretreatment, showing a significant difference in overall survival (p = 0.022) and progression free survival (p = 0.001).

The average pretreatment ADC value was 1.21 *10–3 mm2/s and the average pretreatment T2* value was 30.2 ms. The pretreatment ADC value was a significant predictor of both PFS and OS: a 0.1 *10–3 mm2/s higher pretreatment ADC value provided a hazard ratio of 0.749 for progression (95% CI 0.561–1.000, p = 0.05) and a decreased risk of dying (HR 0.667, 95% CI 0.466–0.955, p = 0.03). Kaplan Meier survival plots for the patients with higher and lower than median ADC are shown in Fig. 2.

Pretreatment T2* value was a significant predictor for OS, but not for PFS. A 1 ms higher average T2* time was associated with an increased risk of dying (HR 1.118, 95% CI 1.023–1.222, p = 0.01).

Combining the pretreatment ADC values and SUV_max_ in a multivariate model showed a higher predictive value for survival, p<0.01. Adding T2* or TLG parameters did not increase the predictive value of the model.

Pretreatment SUV_max_, ADC and T2* were not correlated to the size change on CT after three cycles of chemotherapy, neither in the patient-by-patient analysis nor in the lesion-by-lesion analysis.

### Predictive value of early changes in ^18^F-FDG PET and MRI parameters

SUV_max_ significantly decreased one week after start of treatment (SUV_max_: 11.3 to 6.3, p<0.01). SUV_max_ decreased on average with 18.0% ±19.1% (range 66.1% decrease-14.8% increase).TLG decreased on average 19% ±35.0% after one week of treatment (1084 to 726, p = 0.07). The ADC values significantly increased after one week of treatment from 1.20 to 1.27 *10–3 mm2/s (p = 0.01). The average increase was 0.0710–3 mm2/s ± 0.13*10–3 mm2/s. The T2* values were on average 30.2ms before treatment and 26.7ms one week after start of treatment. This was not a significant decrease (p = 0.17).

**Fig 3:**
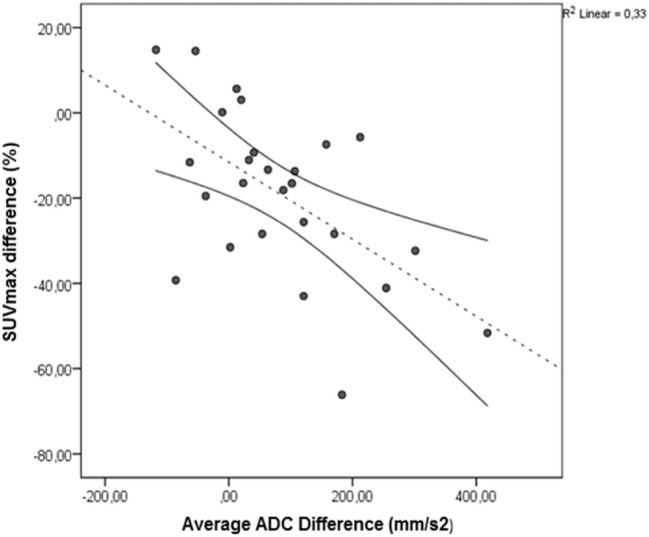
Regression analysis of the changes in SUV_max_ on differences in ADC one week after start of treatment (r = -0.58, p = 0.002). The fit line (red) and 95% confidence interval (black) are shown.

The change in ADC value was inversely correlated to the change in SUV_max_, r = -0.58, p<0.01, Fig. 3. No correlation with changes in T2* was observed. The degree of change in SUV_max_, TLG and ADC were not predictive of either PFS or OS. Also combined changes in SUV_max_, TLG and ADC did not predict PFS or OS.

**Fig 4:**
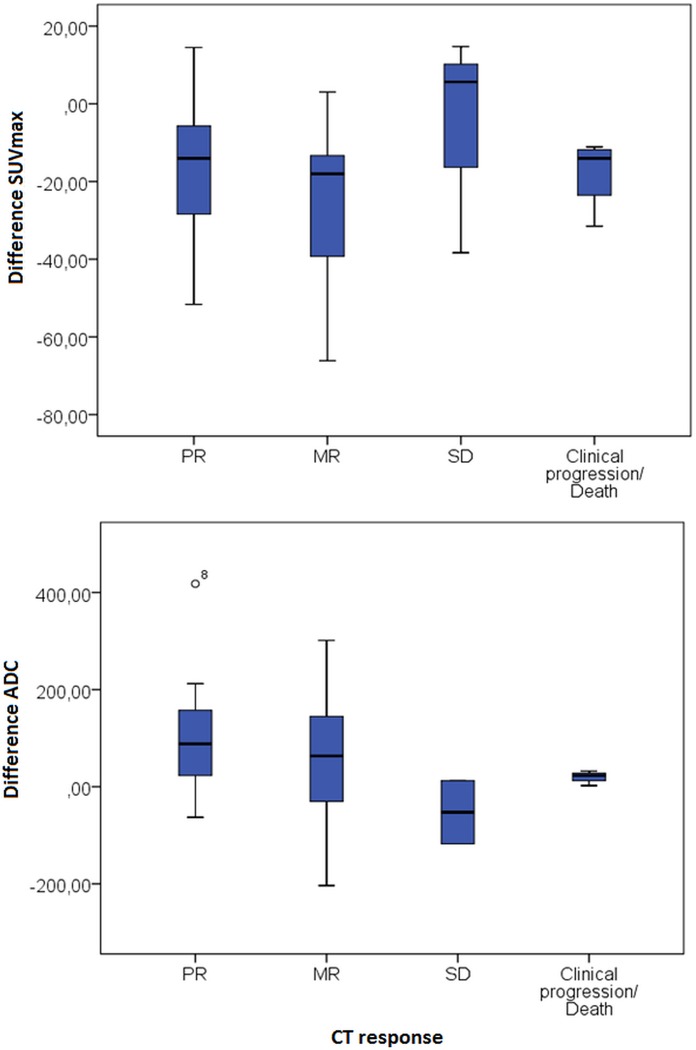
Boxplots showing the change in SUV_max_ and ADC in patients with partial response, minor response, stable disease and in patient unable to perform the scan after 3 cycles due to clinical progression or death. There was no relation between response and changes in SUV_max_ or ADC.

Furthermore, the change in SUV_max_, TLG and ADC after one week did not correlate to size change on CT after 3 cycles in a lesion by lesion analyses or patient-by-patient analysis. As shown in Fig. 4, there is no difference between CT responders and non responders in change in SUV_max_ or ADC. Of note, in only 23 metastases an increase in SUV after one week of treatment was observed, of which 16 metastases had a reduced diameter after 3 cycles.

## Discussion

### Pretreatment ^18^F-FDG PET and MRI parameters

Combining the pretreatment ADC values and SUV_max_ in a multivariate model showed a higher predictive value for survival. Also, a correlation between SUV_max_ and survival was observed. This is in line with previous studies, performed in patients with both primary resectable and irresectable colorectal liver metastases, in which significantly higher pretreatment SUV was associated with poorer outcome [7,22].

In this study, lower pretreatment ADC values predicted poorer outcome, which may be related to the association between ADC values and histological grade. In breast and prostate cancer lower ADC values were correlated with higher histological grade and higher Gleason score [23–25]. In colorectal cancer, a higher histological grade is correlated with an adverse outcome [26,27]

A low T2* value, indicating higher concentration of deoxyhemoglobin, was favorable for longer OS, but did not correlate with PFS. However, the deoxyhemoglobin is not only dependent on hypoxia. In very poor vascular supply and necrotic areas, there may be also very little deoxyhemoglobin present.

### Early changes in ^18^F-FDG PET and MRI parameters

Both ^18^F-FDG PET and DWI showed therapy induced changes one week after start of treatment. The changes in ^18^F-FDG PET and DWI were correlated, suggesting an association between treatment effects leading to decreased cellular density and metabolic response.

Unfortunately, the measured treatment effects were not predictive of survival or CT-response. The lack of (strong) relation between changes in ^18^F-FDG PET and MRI parameters and treatment outcome can be attributed to several factors. In contrast to comparable previous studies, bevacizumab was part of the standard treatment regimen in the present study. Antiangiogenic treatments, such as bevacizumab, may alter the distribution of intravenously injected tracers or contrast agents in the tumor [28]. Decrease in SUV might therefore, in part, reflect alterations in tumor blood supply, rather than metabolic changes. This might obscure the association between metabolic response and treatment outcome. The use of a variable threshold to delineate the tumor on ^18^F-FDG-PET may have resulted in an underestimation of the effect of treatment on TLG, since SUV_max_ decreased as an effect to treatment and therefore the threshold was lower for the second scan. However, the use of a fixed threshold is also problematic due to high background activity in liver tissue.

DWI and T2* are not dependent on administration of intravenous contrast agents. However, the observed increase in ADC and reduction in T2* may be a reflection of therapy induced necrosis, related to a favorable outcome, as well as to bevacizumab induced reduction in vascularization and hypoxia, related to therapy resistance and a dismal outcome. Thus, the relation between outcome and changes in DWI and T2* are obscured by differential effects on the tumor. T2* did not significantly alter after start of treatment. Therefore, this parameter may be less suitable as a predictor of response to therapy.

Finally, the interval between start of treatment and the first evaluation may be of crucial importance for response monitoring with 18F-FDG PET and ADC. An interval of one week after start of targeted therapy containing treatment might be too short for 18F-FDG PET. 1–2 weeks after start of treatment a flare-up phenomenon has been described, defined by an initial rise in metabolic activity in lesions that would respond later on [29]. This phenomenon might also interfere with the measured parameters.

Conversely, changes in ADC cannot only precede changes in tumor size, but may even disappear after a certain time due to activated repair mechanisms [30]. In a previous study an increase in ADC in colorectal and gastric liver metastases (of 23 patients) was described after 3 and 7 days of treatment. However, only a weak correlation between change in ADC on day 3 and final change in size could be established, whereas no significant correlation between changes at day 7 and final lesion size was observed [13]. Therefore, it might be more effective to measure ADC at a shorter time interval after start of treatment. Finally, we can only speculate on the optimal interval for early response prediction using T2* in the present study. In a breast cancer study after 2 cycles of chemotherapy, however, a significant increase in T2* was observed [31], suggesting that longer time intervals may benefit.

### Study limitations

Due the limited number of patients we can conclude that there is no high prognostic value in the early changes in PET-values and ADC after start of treatment, but weaker relations (with lower prognostic value) could be easily missed.

The patients with the poorest treatment outcome could not complete all scans which further limits correlation with changes in size after 3 cycles. The use of two different PET-CT scanners should not have influenced the predictive value of the pretreatment SUV and TLG measurements as both scanners were EANM-EARL accredited for quantitative PET.

## Conclusion

Pretreatment TLG values, ADC and T2* in colorectal liver metastases are predictive of patient survival. In the future, this could provide the opportunity to select patients who could benefit from chemotherapy prior to start of treatment. Early effects after one week of treatment with chemo- and targeted therapy can be measured with ^18^F-FDG PET and DWI, however, these effects were not predictive of long term outcome in this study.

## Supporting Information

S1 Dataset This excel file contains the (anonymous) raw data needed to reproduce our results. All patients were given one study number and have scan data in up to three time points: before chemotherapy (1), one week after chemotherapy (2) and after 3 cycles (3). Average T2* of all assessed lesions (in ms) is given, as is the difference in T2*. The mean ADC per assessed tumor and the average ADC of all assessed tumors is given (in *10–6 mm/s2). The SUV_max_ per tumor, the volume-weighted average SUV_max_ and the total TLG are given. The maximum diameter (in mm) per assessed lesion is reported, as is the calculated response on CT. Furthermore, it reports whether patients had a metastasectomy after study participation (2) of not (1), were progressive (1) during follow up or had still a response (2), died during follow-up (1) or were still alive (2) at the end of follow up. Progression free survival and overall survival are reported in weeks.
(XLSX)
